# Ovarian stimulation in patients with breast cancer

**DOI:** 10.3332/ecancer.2015.504

**Published:** 2015-02-03

**Authors:** Elkin Muñoz, Naira González, Luis Muñoz, Jesús Aguilar, Juan A García Velasco

**Affiliations:** 1 IVI Vigo, Plaza Francisco Fernández del Riego, 7, Vigo 36203, Pontevedra, Spain; 2 Fundación Universitaria de Ciencias de la Salud, Bogotá, Cundinamarca 11001000, Colombia; 3 IVI Madrid, Universidad Rey Juan Carlos, Fuenlabrada, Madrid 28023, Spain

**Keywords:** breast cancer, controlled ovarian stimulation, fertility preservation, letrozol

## Abstract

Breast cancer is the most prevalent malignancy among women under 50. Improvements in diagnosis and treatment have yielded an important decrease in mortality in the last 20 years. In many cases, chemotherapy and radiotherapy develop side effects on the reproductive function. Therefore, before the anti-cancer treatment impairs fertility, clinicians should offer some techniques for fertility preservation for women planning motherhood in the future.

In order to obtain more available oocytes for IVF, the ovary must be stimulated. New protocols which prevent exposure to increased estrogen during gonadotropin stimulation, measurements to avoid the delay in starting anti-cancer treatment or the outcome of ovarian stimulation have been addressed in this review.

There is no evidence of association between ovarian stimulation and breast cancer. It seems that there are more relevant other confluent factors than ovarian stimulation. Factors that can modify the risk of breast cancer include: parity, age at full-term birth, age of menarche, and family history.

There is an association between breast cancer and exogenous estrogen. Therefore, specific protocols to stimulate patients with breast cancer include anti-estrogen agents such as letrozole. By using letrozole plus recombinant follicular stimulating hormone, patients develop a multifollicular growth with only a mild increase in estradiol serum levels.

Controlled ovarian stimulation (COS) takes around 10 days, and we discuss new strategies to start COS as soon as possible. Protocols starting during the luteal phase or after inducing the menses currently prevent a delay in starting ovarian stimulation.

Patients with breast cancer have a poorer response to COS compared with patients without cancer who are stimulated with conventional protocols of gonadotropins.

Although many centres offer fertility preservation and many patients undergo ovarian stimulation, there are not enough studies to evaluate the recurrence, breast cancer-free interval or mortality rates in these women.

## Introduction

Breast cancer is the most common neoplasia among women under 50. It is estimated that during 2012, 1.67 million new cases of breast cancer were diagnosed worldwide; 25% of all cancers, according to WHO data. This percentage is somewhat higher in industrialized countries [1, 2].

Breast cancer is the most prevalent neoplasia type in women of reproductive age. In Europe, the incidence of breast cancer in premenopausal women is 30/100,000 [3, 4]. While in the United States it is estimated that one in every 202 women will develop an invasive breast cancer before the age of 39 [2].

Progress made in the early diagnosis and treatment enables an increasingly higher number of women to overcome breast cancer. In 2009, the risk of death from cancer, of any type, in the United States was estimated at 20% lower than in 1991 [3]. The reduction in mortality makes patients look forward to fulfill other objectives once they overcome the disease. Fertility is one of those objectives which concerns patients who survive cancer.

Cancer treatments affect reproduction in women. The adverse effect that chemotherapy and radiation therapy have on fertility is well known. Initial studies demonstrated follicular depletion and the functional alteration of the ovary [5, 6], although the result is not always ovarian failure. In some cases, treatments are associated with the recovery of menstrual cycles, but with reproductive problems during the long-term. Thus, the implementation of *in vitro* fertilization and embryo cryo-preservation shortly after the treatment may involve some possible genetic risks for the oocytes in development [7]. Breast cancer is the most frequent cause for preserving fertility. It is estimated that approximately half of the women undergoing treatment for fertility preservation are patients with breast cancer [8].

## Chemotherapy in breast cancer

There is evidence that chemotherapy produces an increase in disease-free survival and overall survival of patients with breast cancer. The goal of chemotherapy is to eradicate the micro-metastatic disease responsible for the recurrence and progression of the disease [9, 10].

The administration of six cycles of anthracycline-based chemotherapy reduces the risk of annual death by 38% for women younger than 50, and approximately by 20% for women between 50 and 69 years old. The benefit is independent of the use of tamoxifen, the status of the estrogen receptor, and the nodal status or other characteristics of the tumour. These diagrams, based on anthracyclines, are clearly more effective than other protocol type CMF (cyclophosphamide, methotrexate and fluorouracil) [9, 10]. The addition of taxanes combined with anthracyclines has demonstrated superiority in the survival rate for tumours which are biologically more aggressive [10].

The impact of the treatments for ovarian function depends on the chemotherapeutic agents used, the dose, the age, ovarian reserve, and the association of several drugs or radiotherapy [11, 12].

Patients with stage II breast cancer and those with stage I tumours larger than 1 cm should be warned about the effect of gonad-toxic chemotherapy, since this will form a part of their therapeutic routine [13]. The risk of amenorrhea is not equal for all types of treatments but it varies in relation to the type of chemotherapy and radiotherapy (Table 1).

The addition of taxanes to the treatment schemes based on anthracyclines (AC, FEC, and FAC) is a recommendation of randomized clinical trials [14]. The gonadal toxic effect of taxanes and trastuzumab is not well established, and both are essential drugs in the adjuvant treatment of breast cancer. The addition of taxanes to schema AC (cyclophosphamide, doxorubicin) produces a significant increase in the rate of amenorrhea post-chemotherapy in women over 40, but not in those who are younger [15].

## Association between ovarian stimulation and cancer

There is no absolute evidence of association between ovarian stimulation for IVF and cancer [16]. According to a recent meta-analysis, it has not been possible to associate ovarian stimulation in IVF treatments with the appearance of ovarian, endometrial, or cervical cancer, although the longest period of follow-up in the studies was 10 years [17]. A retrospective cohort study that included more than 20,000 women, with a mean follow-up of 15 years, found a greater number of borderline ovarian tumours in patients undergoing IVF compared with sub-fertile women who did not undergo IVF. This difference was not found for epithelial ovarian cancer [18].

The studies which are looking for the relationship between breast cancer and ovarian stimulation show conflicting results. They are case and control studies with an insufficient number of patients [19, 20]. It seems logical to think that if there were any association between controlled ovarian stimulation (COS) and breast cancer, the tumours would develop after stimulating a woman who should present an over expression of oestrogen receptors (ER). One study examines the histological and molecular characteristics of breast cancers diagnosed until 24 months after COS. When compared with a control group of similar age without exposure to COS, no differences between the two groups were found [21].

## How to stimulate a patient with breast cancer?

There are several options available for preserving fertility in cancer patients: embryo cryo-preservation, vitrification of oocytes, ovarian tissue freezing, and maturation of oocytes *in vitro*. These last two techniques, which are less widely used, are considered experimental despite there being protracted experience with its use [22–24]. The cryo-preservation of embryos is the most widespread method being used and in which there is a greater experience. In recent years, vitrification of oocytes has become a widely used technique [25]. Cryo-preservation of embryos, such as oocytes, is usually carried out after COS.

A conventional IVF cycle requires approximately 10–14 days of COS with gonadotropins to achieve multi-folicular development. This results in an elevation of the serum estradiol, up to 10–15 times higher than physiological levels. To avoid this elevation of estradiol in patients with breast cancer, many protocols have been developed for stimulation with gonadotropins associated with tamoxifen or Aromatase inhibitors (letrozole) [26–32]. The peak of estradiol in the protocols of ovarian stimulation with tamoxifen or with letrozole is slightly higher than the levels achieved in a natural cycle; around 300–350 pg/ml [27].

The most widely used protocol to stimulate patients with breast cancer is the oral administration of letrozole 5 mg from day 2–3 of the cycle, in conditions of ovarian quiescence [follicular stimulating hormone (FSH) < 13 IU/L/E2 < 60 pg/ml]. After 2 days of treatment with letrozole, a variable dose of recombinant FSH (rFSH) between 150 and 300 IU/day is added. When the concentration of serum estradiol exceeds 250 pg/ml or the follicles reach a size greater than 13 mm in diameter, administration of GnRH antagonists is started to avoid the premature peak of LH. Follicular growth is monitored until at least two of the follicles reach 20 mm in diameter and at that moment ovulation is triggered with the agonists of GnRH. By comparing the use of GnRH agonists versus hCG trigger ovulation, it was found that the agonists achieved a greater and faster decline of the estradiol levels without reducing the number of mature oocytes collected or the fertilization rate [28, 30].

This protocol with letrozole, along with final rFSH and the induction of ovulation with GnRH agonists (triptorelin), has been implemented in an extended form, independent of the molecular phenotype of breast cancer [26–32].

There is no evidence that the exposure of the oocytes to letrozole increases congenital defects at birth. However, the effect is not known if the embryos are exposed to systemic letrozole [31, 32].

Novartis, the company responsible for the production of Femara (letrozole) does not recommend the use of this drug as an inducer of ovulation. Novartis states that the drug should not be used in women who may become pregnant, during pregnancy and/or breast-feeding, because there is a potential risk of harm to mother and fetus, including risk of fetal malformations. A study of 911 newborns shows similar overall rates of major and minor congenital malformations among newborns from mothers who conceived after letrozole or clomiphene citrate [33]. In Spain, the Ministry of Health, Social Services, and Equality has approved its compassionate use in patients with breast cancer.

## How to avoid a delay in the start of the stimulation?

In oncology, patients are not advised to delay the start of COS. It is therefore usual to begin the stimulation outside days 2–3 of the menstrual cycle. The strategy varies according to the time of the cycle.

In the early proliferative phase, when there is still no dominance follicular, stimulation starts even if the patient is not on day 2–3 of the cycle. If the cycle is in a proliferative phase after the follicular dominance and the follicular size is ≥18 mm in average diameter, the puncture of the follicle and the vitrification of the oocyte are performed. Subsequently, the GnRH antagonist is applied for 5 days. If the dominant follicle is less than 18 mm, it is stimulated with minimal doses of FSH, until the follicle reaches 18 mm in diameter and follicular puncture is performed. The GnRH antagonist is then administered for 5 days.

When the patient is in the secretory phase of the cycle, the ovulation is confirmed by ultrasound along with the analytical determination of estradiol and progesterone. The GnRH antagonist is administered for 4–5 days, and then stimulation is begun. The objective of the antagonists of GnRH is to achieve estradiol levels below 60 pg/ml and not to delay treatment until the start of a new physiological cycle [28, 30, 34, 35].

In some cases, the administration of gonadotropins had been attempted during the secretory phase to simulate the appearance of a second wave of endogenous gonadotropins that occur in a physiological way. However, ovarian stimulation which is started during the secretory phase is a strategy which has variable results [35].

## Responses to ovarian stimulation in cancer patients

Cancer is associated with increased states of catabolism and malnutrition. Many patients experience weight loss, which could affect the hypothalamic-pituitary axis and lead to a reduction in reproductive capacity. The occurrence of psychological stress could lead to an increase in prolactin and endogen opioids. Further to this, levels of gonadotropins could decrease, thus affecting fertility [36]. Therefore, illness could affect the ovary and its responsiveness, even prior to chemotherapy and radiotherapy [37, 38].

Studies of ovarian reserves in those whose Anti-Mullerian Hormone (AMH) levels have been measured show that women with cancer have lower levels of AMH prior to receiving chemotherapy than the expected values according to their age group [39]. The antral follicle count is also lower in women with cancer compared to a healthy age-matched control group [40]. Recently, some studies point to the possible association of BRCA1 mutation with the reduced ovarian response to stimulation [41].

According to these considerations, various different studies have compared the result of COS in cancer patients and cancer-free IVF patients. A recent meta-analysis concludes that women with cancer who undergo COS in order to maintain fertility during cancer treatment produce fewer oocytes than healthy patients of similar age [38]. Other findings include a longer duration of COS and higher levels of gonadotropins in cancer patients [34].

Our group has demonstrated that women with breast cancer produce 2.4 fewer oocytes than women of a similar age stimulated via a conventional IVF protocol [36]. No prospective randomized studies have currently taken place which compare clomiphene citrate or tamoxifen and gonadotropins with a conventional protocol consisting only of gonadotropins [40].

Other groups produced different results. Oktay showed that the number of oocytes and embryos obtained in a group stimulated using letrozol was similar to that of patients stimulated using other protocols [27–31].

## Is it safe to simulate patients with cancer?

Few studies have evaluated the potential risks of stimulating the ovaries in breast cancer patients. Following a protocol of letrozol plus FSH and inducing ovulation with GnRH agonist ensures low levels of oestrogen, which reduces concern over the safety of COS.

Up to now, differences in survival and recurrence of breast cancer rates have not been observed in patients who underwent COS compared with those who did not. The studies present certain limitations in methodology, such as the insufficient size of the sample group and the short duration of the monitoring [43–45]. One study monitored participants for varying lengths of time (between 272 and 600 days) post COS. No differences were found in the recurrence of breast cancer when compared with those patients who did not undergo COS [31].

Breast cancer patients are usually referred for chemotherapy 6–8 weeks after surgery. However, in the early stages of cancer, the chemotherapy treatment can be delayed until 12 weeks without affecting the prognosis, but the patients need to be referred early for diagnosis by a fertility specialist [46]. It is possible to carry out two cycles of COS in the intervening time between surgery and the start of chemotherapy. This strategy garners the best results in terms of the number of eggs and embryos without demonstrable difference in recurrence rates [43]. Generally, it can be stated that COS does not delay the start of chemotherapy, but the patient needs to be referred early for diagnosis by a fertility specialist. It is also necessary for treatment to preserve fertility to be authorised by the oncologist overseeing the patient’s treatment.

Neither the cancerous nor the normal cells in the breast react to the gonadotropins (FSH, LH) nor to hCG [45]. Conversely, there is cellular proliferation and an increase in cancer cell lines ER^+^ (oestrogen receptor) with exposure to oestrogen, and it is dose-dependent. Exposure to progesterone or clomiphene citrate shows a decline in cellular proliferation in the ER^-^ line, with a mutation of BRCA1. On the other hand, the hCG shows a tendency to protect [48]. These findings support the idea that COS with minimal elevation in levels of estradiol does not present an added risk for breast cancer.

## Conclusion

Preserving fertility is an option for many women who suffer from breast cancer. Freezing embryos and vitrification of oocytes are the two most common options for treatment and both require previous COS. The COS does not represent a risk for breast cancer. The stimulation protocols using anti-oestrogen agents have proven effective, with a minimal increase in estradiol levels. There are strategies to induce baseline ovarian conditions using GnRH antagonists, avoiding a delay in starting ovarian stimulation. Ovarian reserves and response are lower in women with breast cancer. Well-designed studies are required to evaluate the long-term safety, survival rates, and remission period after ovarian stimulation in women with breast cancer.

## List of abbreviations

IVF: In Vitro Fertilisation
hCG: Human Chronic Gonadotropin
FSH: Follicle Stimulating Hormone
GnRH: Gonadotrophin Releasing Hormone
LH: Luteinizing Hormone
COS: Controlled Ovarian Stimulation
ER: Oestrogen Receptors
PR: Progesterone Receptors
AFC: Antral Follicle Count
FAC: Fluorouracil Adriamycin (Doxorubicin) Cyclophosphamide
FEC: Fluorouracil Epirubicin Cyclophosphamide
CMF: Cyclophosphamide Methotrexate Fluorouracil
AC: Adriamycin (Doxorubicin) Cyclophosphamide
TAC: Taxotere (Docetaxel) Adriamycin (Doxorubicin) Cyclophosphamide
TCH: Taxotere (Docetaxel) Carboplatin Herceptin (trastuzumab)

## Conflicts of interest

No conflict of interest.

## Figures and Tables

**Table 1.** Risk of permanent amenorrhea in women treated with chemotherapy and radiotherapy.

High Risk > 80%	External radiotherapy that includes the pelvic region CMF, CEF, CAF x 6 cycles. Women > 40 years old (CMF: cyclophosphamide, methotrexate and fluorouracil CEF: cyclophosphamide, epirubicin, fluorouracil CAF: cyclophosphamide, doxorubicin, fluorouracil)
Intermediate Risk	CMF, CEF, CAF x 6 cycles. Women 30–39 years old AC x 4 in women > 40 years (Doxorubicin/cyclophosphamide)
Low Risk < 20%	CMF, CEF, CAF x 6 cycles in women < 30 years old AC x 4 in women < 40 years old
Very Low Risk or no risk	Vincristine Methotrexate Fluorouracil
Unknown Risk	Taxanes Oxaliplatin Irinotecan Monoclonal Antibodies (trastuzumab, bevacizumab and cetuximab) Tyrosine-Kinase Inhibitors (ertolinib, imatinib)

Table is based on ASCO recommendations on fertility preservation in cancer patients [11]
